# Stabilizing Agents for Drug Nanocrystals: Effect on Bioavailability

**DOI:** 10.3390/pharmaceutics8020016

**Published:** 2016-05-20

**Authors:** Annika Tuomela, Jouni Hirvonen, Leena Peltonen

**Affiliations:** Division of Pharmaceutical Chemistry and Technology, P.O. Box 56 (Viikinkaari 5 E), University of Helsinki, 00014 Helsinki, Finland; annika.tuomela@gmail.com (A.T.); jouni.hirvonen@helsinki.fi (J.H.)

**Keywords:** bioavailability, drug nanocrystals, polymers, stabilizer, surfactants

## Abstract

Drug nanocrystals are a versatile option for drug delivery purposes, and while the number of poorly soluble drug materials is all the time increasing, more research in this area is performed. Drug nanocrystals have a simple structure—a solid drug core is surrounded by a layer of stabilizing agent. However, despite the considerably simple structure, the selection of an appropriate stabilizer for a certain drug can be challenging. Mostly, the stabilizer selection is based purely on the requirement of physical stability, e.g., maintaining the nanosized particle size as long as possible after the formation of drug nanocrystals. However, it is also worth taking into account that stabilizer can affect the bioavailability in the final formulation via interactions with cells and cell layers. In addition, formation of nanocrystals is only one process step, and for the final formulation, more excipients are often added to the composition. The role of the stabilizers in the final formulation can be more than only stabilizing the nanocrystal particle size. A good example is the stabilizer’s role as cryoprotectant during freeze drying. In this review, the stabilizing effect, role of stabilizers in final nanocrystalline formulations, challenges in reaching *in vitro–in vivo* correlation with nanocrystalline products, and stabilizers’ effect on higher bioavailability are discussed.

## 1. Introduction

**Figure 1:**
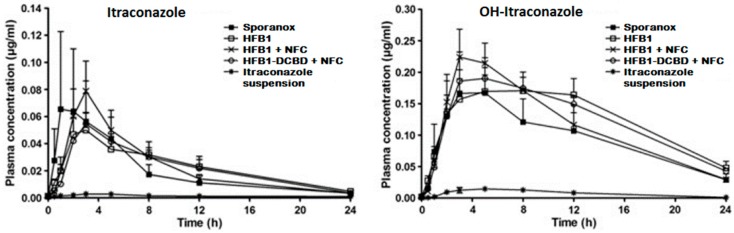
Plasma concentration–time profiles of itraconazole (**left**) and OH-itraconazole (**right**) in rats after oral administration of Sporanox^®^, three different hydrophobin coated nanocrystal formulations and itraconazole microsuspension in rats. AUC values for nanocrystal formulations were 1.2–1.3-fold higher than with Sporanox^®^ (reprinted from [6] with permission. Copyright Elsevier 2011).

It has been approximated that poor drug solubility is an issue for approximately 70%–90% of new drugs [1,2]. Poorly soluble drugs can be divided further into two classes: (i) grease ball and (ii) brick dust molecules [3]. Grease ball molecules, despite poor water solubility, are soluble to lipids, e.g., insolubility is due to the solvation extreme, and can be formulated, for example, to lipid formulations. Brick dust molecules are poorly soluble due to crystal packing interactions being insoluble to both aqueous solvents and lipids, and they benefit, for example, on the formation of amorphous materials or particle size decrease, e.g., nanocrystallization. As an example of a brick dust molecule, the solubility of AZ68, a neurokinin NK receptor antagonist intended for schizophrenia treatment, was increased by both producing amorphous nanosuspension and nanocrystalline formulation, and a solution with solubility enhancing excipients PEG and DMA was used as a reference sample [4]. AZ68 has low solubility in the gastrointestinal track but high permeability, hence being a BCS class II compound. AZ68 was absorbed slower from crystalline nanosuspensions (longer *t*_max_), but the total absorbed amount of the drug was similar to all the formulations. In another study with itraconazole, *in vitro* drug nanocrystals had superior drug release, but, *in vivo*, they failed as compared to Sporanox^®^ due to the precipitation and fast transit time from the stomach to the intestines [5]. When the itraconazole nanocrystals were packed inside of a cellulose matrix, the AUC values as compared to Sporanox^®^ were 1.2 to 1.3 times higher [6] (Figure 1). Accordingly, nanocrystals are in many cases a good option for drug delivery purposes, but further formulation steps present a critical role for successful drug release profiles *in vivo*.

Drug nanocrystals are nanosized solid drug particles surrounded by a stabilizer layer. Often, nanocrystals are considerably easy to produce, but the stability and the selection of stabilizer(s) is the most challenging and critical step [7]. Stabilizers stabilize the newly formed drug nanocrystals, but they also have an important role in further formulation and they even affect drug bioavailability, which should be kept in mind when making stabilizer selections. For example, Beirowski *et al.* studied the effect of stabilizers and added cryoprotectants to improve the stability of drug nanocrystals during freeze-drying [8]. They noticed that if the stabilizer concentration and hence the steric stabilizer layer around the particles was dense enough, nanocrystals were separated from each other during the drying, and the formation of van der Waals forces was not possible between the particles. In this case, no extra cryoprotection was thus needed during the drying process.

Sharma *et al.* demonstrated the impact of functional stabilizers on the *in vivo* efficiency and bioavailability of paclitaxel nanocrystals [9]. The aim of the study was to enhance the absorption of paclitaxel with multiple pathways, based on the fact that paclitaxel bioavailability *in vivo* is limited by low solubility, rapid metabolism and efflux by P-gp transporters. Pluronic-grafted chitosan copolymer was used as a stabilizer. *In vitro* analysis with a Caco-2 cell line revealed that paclitaxel accumulation inside the cells was higher with nanocrystals as compared to Taxol™ due to the P-gp inhibitory effect of the pluronic-grafted chitosan stabilizer in the nanocrystals. It was also noticed that the stabilizer reversibly opened tight junctions between the cells enabling the paracellular route for drug transport. Final proof was reached in the *in vivo* absorption tests, where 12.6-fold improvement in relative bioavailability was found with nanocrystalline formulation as compared to Taxol™.

As is obvious from the above-mentioned examples, stabilizer selection can dramatically affect the optimized drug nanocrystals’ performance during the further formulation steps and *in vivo*. In this review, the stabilizing agents related to nanocrystal formulations are discussed. First, the functioning of stabilizers and their interactions with drug particles are presented. Then, the most utilized stabilizers and a couple of interesting new materials are described. Finally, the effect of stabilizer selection for the final formulation and possible permeation enhancing effects are reviewed. Although the nanocrystals are stabilized during particle formation, the stabilizer selection should already reflect the needs of the final formulation.

## 2. Stabilizers

Both polymers and surface active agents have been utilized as stabilizers for pharmaceutical nanocrystals. In Table 1, some examples of drugs and stabilizers in drug nanocrystals are presented (Table 1), and, in the following chapters, utilized stabilizers for drug nanocrystals are primarily reviewed.

### 2.1. Poloxamers

Poloxamers are synthetic polymers consisting of a series of closely related block copolymers of ethylene oxide and propylene oxide [18,25,26]. Poloxamers have been explored and applied in the field of pharmacy and biomedicine as thermo-sensitive hydrogels, nanoparticle and micelle carriers, particle surface coatings, and tissue scaffolds [27,28]. The FDA has approved the majority of them as food additives and pharmaceutical ingredients [29]. They are generally considered nontoxic materials and are typically applied in oral, parenteral and topical formulations as polymeric stabilizers. Today, they are especially utilized as stabilizing agents for drug nanocrystals.

Poloxamers are fabricated by adding ethylene oxide to polyoxypropylene glycol, which is a product from the reaction between propylene oxide and propylene glycol. These amphiphilic block polymers, comprised of hydrophobic polypropylene oxide (PPO) and hydrophilic polyethylene oxide (PEO) chains, are regarded as more advantageous nanocrystal stabilizers than, *i.e.*, traditional homopolymers. The most common poloxamers utilized in nanocrystalline formulations are Pluronics^®^, *i.e.*, poloxamer 407 (Pluronic^®^ F127) and poloxamer 188 (Pluronic^®^ F68). Both of these polymeric stabilizers are non-ionic linear triblock copolymers consisting of a hydrophobic central segment of PPO and two hydrophilic side segments of PEO. While the hydrophilic PEO segments surround the drug crystals providing steric hindrance and preventing particle aggregation and growth, the adsorption on the crystal surface is driven by hydrophobic interactions of the hydrophobic PPO chains. Depending on the used drug compound, the F68 may exert less kinetic restriction in the adsorption process and faster diffusion due to its lower molecular weight compared to F127 (8400 g/mol *vs.* 12,600 g/mol) [30]. In addition to the linear PEO–PPO–PEO type poloxamers, there exists reverse structured poloxamers, *i.e.*, Pluronic^®^ Reverse 17R4, having a reverse structure as compared to Pluronics^®^. 17R4 presents a block copolymer with terminal secondary hydroxyl groups, and is also called a telechelic polymer (PPO–PEO–PPO). However, the linear PEO–PPO–PEO type structures are generally reported as more efficient in nanocrystal stabilization, since the telechelic polymer structure of 17R4 may promote inter-particle bridging and aggregation after nanocrystallization [18].

Besides the stabilizer selection, several factors affect the particle size and stability of nanocrystal suspensions: molecular weight, functional groups, morphology of polymers and hydrophilic/hydrophobic ratio in the stabilizer molecule. The driving force for poloxamer diffusion and absorption onto the crystal surface is the hydrophobic moiety of copolymer; high hydrophobicity of polymers facilitate fast diffusion, strong absorption and sustained time for desorption of polymer. Compared to the amphiphilic polymers and surfactants, hydrophilic polymers are generally less efficient stabilizers for nanocrystals of poorly soluble drugs, due to the absence of the thermodynamic driving force to adsorb on the hydrophobic drug surfaces [31]. Additionally, amphiphilic polymers can decrease the interfacial tension and increase the wettability of nanocrystals. The poloxamers facilitate a decrease in zeta potential with increasing molecular weight (poloxamer F68 < F127). The decline in zeta potential suggests a formation of a sterically stabilized polymer layer. The decrease is even highlighted by increasing poloxamer concentration, especially with poloxamer F127.

**Figure 2:**
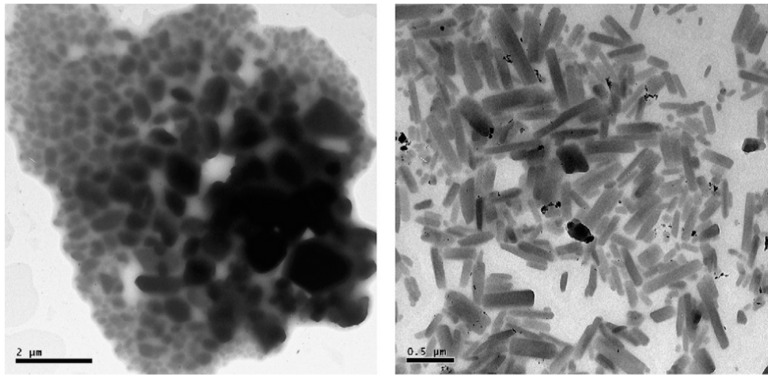
TEM images of poloxamer F127 stabilized indomethacin (**left**) and itraconazole (**right**) nanosuspensions. Nanocrystals are produced by milling (reprinted from [25] with permission. Copyright Elsevier 2011).

Accordingly, the successful stabilizer selection is strongly dependent on the used drug compound. Despite the advantageous and versatile nature of the poloxamers, they are not effective for all drugs. As described earlier, their attachment onto particle surfaces is based on physisorption; if hydrophobic/hydrophilic forces are not strong enough for surface absorption, they are ineffective. Even though poloxamers (F68 and F127) and polysorbate 80 had been proven effective in stabilizing indomethacin and itraconazole nanocrystal suspensions [18,25,32] (Figure 2), they were unsuccessful in the production of brinzolamide nanocrystal suspensions by the wet ball milling technique; the resulting material was micron-sized (>1000 nm) and polydispersed with all the studied stabilizers [21].

Finally, for instance, an application of a combination of ionic surfactants with polymeric stabilizers like poloxamers may provide an enhanced stabilization, which combines the advantages of both the electrostatic and steric stabilization. When poloxamer F68 was combined with small quantities of chitosan derivatives to improve the stability of nanocrystals, an enhanced stability of itraconazole nanocrystals was obtained when compared to F68 alone [33].

Danhier *et al.* compared poloxamer stabilized anti-cancer multi-targeted kinase inhibitor MTKi-327 nanocrystalline formulation with other nanocarrier systems, PEGylated PLGA nanoparticles, polymeric micelles, and also to Captisol solution [11]. The particle size of all the nanoformulations were under 200 nm. Injections with all the three nanoformulations with the highest possible dose did not cause any side effects on mice, but the maximum tolerated dose of Captisol solution was 20-fold lower. After *iv* and oral administration, the AUC value was 2.4 times higher with nanocrystalline formulation as compared to the corresponding value with Captisol solution.

### 2.2. Celluloses

Celluloses are polymers of natural origin, which are frequently utilized in pharmaceutical applications and regarded generally as nontoxic and nonirritating substances. One of the most utilized types of cellulose in pharmaceutical nanocrystal applications is hydroxypropyl methylcellulose (HPMC), categorized as a semisynthetic non-ionic polymer. Typical molecular weights of HPMC vary between 10,000 and 1,500,000 g/mol. In order to produce HPMC, an alkali cellulose is treated with chloromethane and propylene oxide, purified afterwards and ground to fine powder. Different molecular weights, meaning basically varying viscosity grades of the polymer, can be obtained by exposing HPMC to anhydrous hydrogen chloride in order to induce depolymerization. As a hydrophilic polymer, HPMC is used widely in oral controlled drug delivery systems [34]. HPMC is also approved for ocular drug delivery as an inactive ingredient and has been used as an ophthalmic lubricant and tear substitute [35,36]. The beneficial characteristics of HPMC, such as low toxicity, prolonged contact with the mucosa and, most of all, a highly swellable nature, conferred beneficial effects on the release kinetics of the incorporated drug, favoring the use of HPMC in the formulations of ophthalmic drug delivery systems [35,37,38]. In addition, HPMC has lubricant and ocular wound healing accelerating properties. Typically, in nanocrystalline applications, HPMC is applied as a stabilizing agent, using the lowest molecular weights of HPMC [21,23].

Fenofibrate nanocrystal tablet formulations were successfully produced by ball milling technique using HPMC combined with SDS as a stabilizer [39]. The tablet formulations maintained the advantageous properties of nanocrystals regarding particle size, dissolution profile and bioavailability as compared to a commercial product.

**Figure 3:**
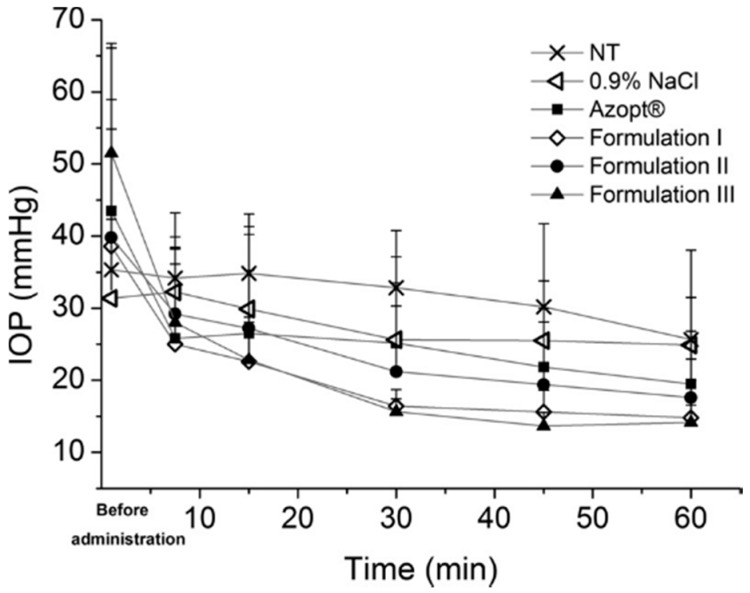
The intraocular pressure (IOP) values as a function of time after topical application of brinzolamide nanocrystal Formulations I–III, Azopt, 0.9% NaCl and the non-treated group (NT). Nanocrystal Formulation III was most efficient in reducing the intraocular pressure (reprinted from [21] with permission. Copyright Elsevier 2014).

More recently, HPMC was applied in an ocular drug delivery system as a stabilizer for ophthalmic, intraocular pressure reducing brinzolamide nanocrystal suspension formulations [21] (Figure 3). Nanocrystal formulations including the HPMC as a stabilizer were prepared at pH 7.4 and pH 4.5 using the wet milling technique. Additionally, an absorption enhancer, polysorbate 80, was included to promote the drug action. The HPMC amount of 25% *w*/*w* in relation to drug amount provided the most stable nanocrystals, whereas the 10% *w*/*w* HPMC concentration was inadequate for providing small and even particle size fractions (620 nm/PI 0.40 *versus* 640 nm/PI 0.51). As mentioned already before, attachment of HPMC on particle surfaces is mostly based on hydrogen bonding. The molecular structure of HPMC contains a high degree of substitution of the methoxy and hydroxypropoxy groups, which can effectively attach onto the brinzolamide nanocrystal particle surfaces via hydrogen bonding [40]. With its relatively high molecular weight, polymeric HPMC provided an effective steric stabilization for brinzolamide [41]. Overall, the intraocular pressure decreasing effect with the prepared formulations appeared to be remarkable in hypertensive rats [21]. However, the effect was the most significant and extended with the formulation buffered to pH 4.5 (in order to maximize the amount of the free drug in the formulation, Figure 3, Formulation III). The results are most likely based on the nanosize of the drug particles and the mucoadhesive properties of HPMC [37,42,43].

In addition to HPMC, other semisynthetic non-ionic polymers are also utilized as nanocrystal stabilizers; hydroxypropylcellulose, (HPC, approx. 50,000–1,250,000 g/mol), hydroxyethylcellulose (HEC), and methylcellulose (MC, approx. 10,000–200,000 g/mol), are ethers of pure cellulose, where some of the cellulose hydroxyl groups are hydroxypropylated (HPC), hydroxyethylated (HEC) or methylated (MC), correspondingly. HPC and MC are used in oral and topical applications, whereas HEC is applied in ophthalmic and topical drug delivery. These homopolymers always contain hydrophilic backbone chains. These polymers can adsorb on the particle surface by hydrogen bonds to form a hydrodynamic boundary layer. As described, cellulose ethers (HPMC and MC) contain a high degree of substitution as methoxy or hydroxypropoxy groups, which can form hydrogen bonds with the drug and inhibit the crystal growth. This degree of substitution determines the stability of the nanocrystal suspensions. For instance, hydrophobic drug surfaces without polar functional groups may be ideal for HPC to physically adsorb and produce steric stabilization, since the hydrogen bonding between the polymer and drug tends to interfere with the stabilization activity of polymers [44]. The efficiency of HPC (38.5% *w*/*w* in relation to the drug amount) as a nanocrystal stabilizer has been demonstrated with 7 different drugs: the smallest nanocrystals were 70 nm in size with anthracene as a drug material and 120 nm with naproxen as a drug material. Moreover, eight different drugs were nanocrystallized with the presence of HPC (particle sizes from 229 to 449 nm) [30]. Again, the importance of the hydrophobic part of the polymer was addressed as a driving force for efficient surface adsorption.

Finally, the efficiency of HPMC, HPC and PVP as stabilizers for naproxen nanocrystals produced by ball milling was compared; the efficiency of the stabilizers varied according to the viscosities of the stabilizing polymers [45]. With polymeric stabilizers, the molecular weight was related to the viscosity, which must be taken into account: high viscosity of the milled dispersions lowers the milling efficiency [7]. In conclusion, HPMC produced nanocrystals efficiently with good storage stability [45]. The smallest nanocrystals were provided by PVP due to the low dispersion viscosity. However, the process parameters had a strong effect on the properties of the PVP containing products.

### 2.3. Vitamin E TPGS

Vitamin E TPGS is an excipient used in pharmaceutical, but also in nutraceutical and cosmetic applications. Vitamin E TPGS is prepared by the esterification of the carboxylic group of crystalline d-α-tocopheryl succinate with polyethylene glycol 1000 (C_33_O_5_H_54_(CH_2_CH_2_O)n). Vitamin E TPGS has shown improved bioavailability properties of poorly absorbed drugs, vitamins and micro-nutrients by acting as an absorption and permeability enhancer [46]. It has also been utilized in the development of Self Emulsifying Drug Delivery Systems (SEDDS) for poorly soluble drugs as an emulsifier. As a water soluble compound, Vitamin E TPGS has also been used as an efficient source of natural Vitamin E, both for therapeutic purposes and nutrition. In the interest of the pharmaceutical industry, Vitamin E TPGS has physical properties that make it a relevant plasticizer for innovative technologies, such as hot melt extrusion. Pharmaceutical companies have incorporated Vitamin E TPGS mainly in oral dosage forms, but new delivery applications are being investigated via parenteral and topical delivery routes (dermal, nasal, pulmonary). Because of the antioxidant properties, Vitamin E TPGS is very attractive for the cosmetic industry, as already mentioned above.

Vitamin E TPGS is a highly stable form of vitamin E, as it is stable when exposed to oxygen, heat, light, or oxidizing agents [47]. However, it is unstable to alkali. Vitamin E TPGS is known to be a stable excipient with a shelf-life of 4 years when stored in an unopened container at room temperature. Importantly, Vitamin E TPGS is stable under the conditions of heat sterilization.

According to Zhang *et al.*, Vitamin E TPGS presents the advantages of PEG and Vitamin E in application of various nanocarriers for drug delivery, including extended half-life of drugs in plasma and enhanced cellular uptake of the drugs [46]. Vitamin E TPGS has an amphiphilic structure exerted by the lipophilic alkyl tail and the hydrophilic polar head, with a hydrophile/lipophile balance (HLB) value of 13.2 and a relatively low critical micelle concentration (CMC) of 0.02% *w*/*w*. These properties make Vitamin E TPGS an interesting molecular biomaterial when developing various drug delivery systems, including prodrugs, micelles, liposomes and nanoparticles. These novel drug delivery systems are, in turn, able to reach sustained, controlled and targeted drug delivery, as well as to overcome the efflux by multidrug resistance (MDR) proteins as a promoter of oral cancer drug delivery by the inhibition of P-glycoprotein (P-gp) [48,49].

Multidrug resistance (MDR) is one of the key factors in the failure of anticancer chemotherapy. Already in 2006 and 2009, Feng and coworkers published positive results on the delivery of biodegradable poly(lactide) and poly(lactide)-*co*-poly(glycolide)—vitamin E derivative—based nanoparticle formulations for the oral delivery of paclitaxel and docetaxel cancer drugs [48,50]. Later on, e.g., Wang *et al.* designed novel multifunctional camptotechin derivative SN-38 loaded alpha-tocopheryl polyethylene glycol succinate (TPGS)/poly(lactic-*co*-glycolic acid) (PLGA) nanoparticles by a modified solvent extraction/evaporation method: 200 nm spherical particles with smooth surfaces, narrow size distribution, appropriate surface charge, and successful drug-loading into the nanoparticles [51]. Cytotoxicity of the TPGS/PLGA/SN-38 nanoparticles against the MDR cells was increased by 3.6 times compared to the free SN-38. Mechanistically, the TPGS/PLGA/SN-38 nanoparticles improved the uptake of the loaded drug by clathrin-mediated endocytosis, and the intracellular nanoparticles escaped the recognition of P-gp by MDR cells. After the SN-38 was released from the TPGS/PLGA/SN-38 nanoparticles in the MDR cells, TPGS or/and PLGA were hypothesized to modulate the efflux microenvironment of the P-gp pump, the mitochondria and the P-gp domain with an ATP-binding site [51], after which the drug entered the nucleus of the MDR cells and induced the cytotoxic effect.

In drug nanocrystals, Vitamin E TPGS has been widely studied due to the above-mentioned activity *in vivo*. Ge *et al.* formulated ursolic acid nanocrystals via an antisolvent precipitation technique using TPGS1000 as a stabilizer [52]. The optimum drug:stabilizer ratio was found to be 0.4:1. With this composition, very small and homogeneous particles were formed (particle size 127 nm and polydispersity index 0.15). The bioavailability was 27.5-fold and *C*_max_ value was 9-fold higher when compared to bulk drug dispersion. The improved bioavailability was partly explained to be due to the P-gp inhibition effect of TPGS1000 on the intestinal epithelium cells.

### 2.4. Soluplus^®^

Soluplus^®^ (BASF) is a pharmaceutical excipient product comprised of polyethylene glycol, polyvinyl acetate and polyvinyl caprolactame-based graft copolymer (PVAc-PVCap-PEG). Primarily, Soluplus^®^ was developed in order to improve drug solubilization properties of poorly soluble active pharmaceutical ingredients (APIs). Model drug studies of Soluplus^®^ based formulation development have been published in the fields of hot melt extrusion, spray drying, high shear dispersions, electrospinning/electrospraying, microwave radiation, solvent casting, solvent evaporation, ball milling, physical/co-milling blends, and thermal heating applications [53,54]. BASF Corp. developed Soluplus^®^ originally for solid solutions, but as mentioned above, it can be used for example in hot melt extrusion, drug layering and spray drying, and it can be also applied as a binder in wet granulation or a dry binder in direct compression [55]. Advantages of Soluplus^®^ include, for example, high flowability and adjustable extrudability.

Yang *et al.* investigated Soluplus^®^ and HPMC stabilized fenofibrate nanosuspensions [22]. Nanocrystals were produced by the media milling technique. In this case, Soluplus^®^ produced considerably smaller particles (344 nm) than HPMC (642 nm). Soluplus^®^ also stabilized the nanosuspension better. The reason for that was found from weaker Ostwald ripening with Soluplus^®^ when the slower diffusion of Soluplus^®^ micelles entrapped dissolved fenofibrate. Bioavailability studies with a physical mixture of fenofibrate and Soluplus^®^ indicated that Soluplus^®^ altered the membrane permeability in the intestines, which increased also the drug permeation. With both of the nanocrystalline formulations, the plasma-concentration curves showed double peaks, indicating different absorption sites for fenofibrate in GI tract.

A permeation enhancing effect of Soluplus^®^ was also found when Linn *et al.* tested Soluplus^®^ for its capability to improve intestinal drug absorption. BCS class II compounds danazol, fenofibrate and itraconazole were tested both *in vivo* in beagle dogs and *in vitro* in transport experiments across Caco-2 cell monolayers [56]. The three drugs were studied as pure crystalline substances, in physical mixtures with Soluplus^®^, and as solid solutions of the drug in the excipient. In animal studies, increased plasma AUC values were observed for the solid solutions in Soluplus^®^ compared to the respective pure drugs. *In vitro* transport studies confirmed the permeability improving effect of Soluplus^®^ on the absorption behavior across Caco-2 monolayers [56].

Finally, Gadadare *et al.* studied top-down and bottom-up approaches for producing Soluplus^®^ stabilized repaglinide nanocrystals [57]. The nanocrystalline formulations enhanced the bioavailability and showed both faster onset and prolonged duration of drug effects compared to pure drug formulation. The saturation solubility level with Soluplus^®^ stabilized nanocrystalline formulations was even 25.67-fold higher when compared to the pure drug.

### 2.5. Others

**Figure 4:**
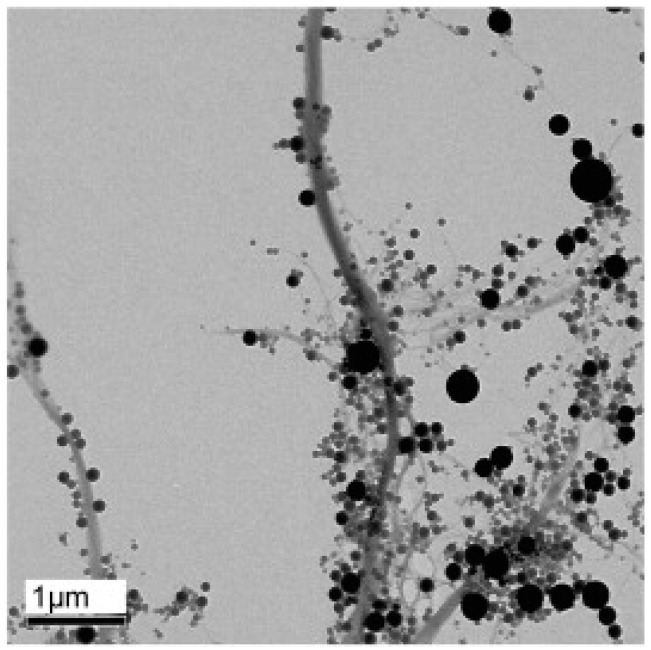
Hydrophobin coated itraconazole nanocrystals attached to a nanofibrillar cellulose network (reprinted from [6] with permission. Copyright Elsevier 2011).

Hydrophobins are surface-active proteins from filamentous fungi, and they can spontaneously form self-assembling monolayers to hydrophobic–hydrophilic interfaces [58]. Valo *et al.* studied hydrophobin as a stabilizer for beclomethasone nanocrystals [58]. With an antisolvent precipitation technique, small nanocrystals were formed, but their long-term stability was not good. In order to improve the long term stability, they coupled two cellulose binding domains to hydrophobin via genetical engineering [6]. Engineered hydrophobin was then utilized as a stabilizer for itraconazole nanocrystals produced by an antisolvent precipitation technique. With the aid of cellulose binding domains, nanocrystals were immobilized to cellulose nanofibrils, which increased their stability (Figure 4). The bioavailability of the nanocrystalline formulation was 1.3-fold higher as compared to commercial Sporanox^®^ (Janssen-Cilag S.p.A., Borgo San Michele, Lazio, Italy). The role of the cellulose material is crucial for drug release profiles. When hydrophobin stabilized, itraconazole nanocrystals were imbedded into red pepper nanocellulose or the microcrystalline cellulose matrix, the *in vitro* drug release was immediate [12], while from bacterial cellulose, quince seed and TEMPO-oxidized birch cellulose matrixes, sustained drug releases were reached [12].

Cyclodextrins are used in pharmaceutical formulations because, via complex formation, they can improve the solubility of poorly water-soluble drugs [59]. In a study with indomethacin, cyclodextrins were used as stabilizers for nanocrystals made by the emulsion solvent diffusion method [15]. The formed indomethacin nanocrystals were 300–500 nm in particle size, and cyclodextrin networks were formed via intermolecular interactions between cyclodextrin molecules stabilizing the particles. Crystal form of indomethacin in the nanocrystals was α-form, while the original bulk drug was of γ-form, which enhanced the dissolution of nanocrystals even more. The dissolution from nanocrystals was fast and complete.

Xia *et al.* formulated PVA stabilized nitrendipine nanocrystals by the precipitation–ultrasonication method [20]. They noticed that after precipitation the new drug crystals were most likely in an amorphous state [60], but when more energy, like ultrasonication, was put into the system, the high-energy amorphous state tended to transform to a more stable crystalline form. Based on XRPD analysis, the crystallinity was increased by ultrasonication [20]. Precipitated nitrendipine formed hydrophobic surfaces, which was fast covered by PVA. However, the crystal growth still continued, because the absorption was not fast enough. However, when the ultrasound was applied to the suspension, the external mass transfer and adsorption rate were intensified. As a result, PVA covered the drug nanocrystal surfaces, and the system reached surface energy and enthalpy minimum values meaning adsorption equilibrium with the sterically stabilized drug nanocrystals. In this case, the ultrasonication after the precipitation process caused the fast change of amorphous form into the more stable crystalline form. At the same time, aggregate formation was hindered with a higher adsorption rate of stabilizer on the nanocrystal surfaces.

Kurakula *et al.* studied cationic charged chitosan as a stabilizer for atorvastatin nanocrystals [61]. Nanocrystals were produced by the probe sonication method. The impact of charge density (different molecular weights of chitosan) on nanocrystal stabilization was studied. The smallest nanocrystals, 394 nm, were reached with low cationic charged type of chitosan. In this case, the stabilization effect was due to both steric and electric stabilization.

## 3. Stabilizing Effect

Formation of nanosized particles creates high energy surfaces, which can turn to aggregation and Ostwald ripening, if stabilization is not at an efficient level. The smaller the particle size, the more important is the efficient stabilization. Stabilization is needed for the formation of nanocrystals as well as for the long-term formulation stability during storage. Drug nanocrystals are formed from a solid core surrounded by a stabilizer layer, and typical stabilizers are amphiphilic surfactants or polymers. Since most of the drug materials that are possible candidates for nanocrystals formulations are poorly soluble and hydrophobic, the amphiphilic stabilizers will also enhance the wetting and dissolution properties of these materials.

Stabilizers can be non-ionic or ionic in nature and the overall stability is based on the classical DLVO-theory reached either via steric hindrance or electrostatic forces. More precisely, stabilizers can be divided into following groups: charged (ionic) surfactants, non-ionic surfactants, polymeric stabilizers and other stabilizers [62]. As already presented, polymers like celluloses (HPMC, HPC, MC, HEC) [13,21,23], PVP [63], Soluplus^®^ [64], poloxamers [18,25], non-ionic surfactants like polysorbates, sorbitan esters, vitamin E TPGS [23,65] or ionic surfactants like SDS [63,66], just to mention a few, have widely been utilized as stabilizers. In single studies, the number of different kinds of stabilizers are numerous and new innovative materials are also tested like hydrophobins [12]. One single stabilizer may be enough, but often combinations of stabilizers are also utilized [67]. In some studies, periodic addition of stabilizers has led to smaller particle sizes and narrower particle size distribution [68]. Periodic addition of stabilizer can be beneficial especially with high viscosity materials. For example, the viscosity can be lowered throughout the process, when only a part of the stabilizer is added to the vessel in the beginning of the process and the rest of the stabilizer is added later in one or more batches during the whole process time.

Steric stabilization is based on the formation of a mechanical barrier, a steric layer, between the particles, and it requires polymeric chains on the particle surfaces that are long enough. Stabilization with non-ionic surfactants and polymers is based on the steric stabilization effect. Temperature changes, for example during the drying, can affect molecular mobility and hence the efficiency of steric stabilization [69]. Electrostatic stabilization is based on formation of repulsive Coulomb forces between the charged colloidal particles. With charged surfactants, the stabilization is mainly based on electrostatic effect. Electrostatic stabilization is sensitive against changes in pH or ionic strength *etc.*, like in the varying conditions of the GI tract, and drying. Electrosteric stabilization is based on a combination of steric and electrostatic effects—for example, stabilization with higher molecular weight charged surfactants.

The selection of the stabilizer is mostly empirical and based on the former experiences of the formulation scientist. The factors determining the stabilization effect are not totally explained [7,13,23], but when considering the stabilization of drug nanocrystals, it is not only the interaction forces, being either hydrophilic/hydrophobic, hydrogen bonding, ionic or some other weak forces, between the drug surface and stabilizer molecule, which need to be taken into account. In addition, a chain length long enough for creating a steric barrier or a high enough zetapotential, absolute values above 30 mV, for the creation of electric barrier, are crucial [18].

Rachmawati *et al.* produced curcumin nanocrystals with PVP, PVA, TPGS, SDS and NaCMC as stabilizers [19]. Stabilizing mechanisms were compared; while PVP, PVA, TPGS are steric stabilizers, NaCMC is both a steric and electrostatic (electrosteric) stabilizer and SDS is mostly a electrostatic stabilizer. This was also confirmed with zeta-potential measurements; SDS has a very high zeta-potential value (below −50 mV), NaCMC was the next highest and PVA, PVP and TPGS had quite low values. Nanocrystals were produced with premilling followed by a high-pressure homogenization technique. SDS stabilized the nanocrystals effectively. TPGS was even better and nanocrystals with narrow particle size distribution were produced after a very short process time. TPGS in solution has low viscosity and high surface activity, which makes it an efficient stabilizer. The higher molecular weight polymers, PVA and NaCMC, required an increased number of homogenization cycles for smaller particle sizes. PVP with lower molecular weight formed smaller particles faster. The poorest stabilizer in this study was NaCMC with the smallest particle size, it being only as high as 824 nm.

Recently, surface plasmon resonance (SPR) and contact angle techniques were successfully applied as tools for monitoring stabilizer–drug interactions and stabilizer affinity for nanocrystal surfaces [18]. Five structurally different types of poloxamers were used as stabilizers for indomethacin nanocrystals. Poloxamer 17R4, with a telechelic structure, formed a loop on the crystal surface. The short PEO length and micellar bridging caused serious agglomeration during the milling. Poloxamer L64 formed a long train-short tail type conformation on the drug surface, which was not adequate to protect the nanocrystals from aggregation. Finally, the F68 folded in half to anchor onto the drug surface through the PPO group, while the long PEO tails made a thick steric layer, which efficiently stabilized the nanocrystals. The L64 exhibited a higher affinity to the indomethacin surface compared to the F68, having, however, shorter hydrophilic PEO chains (approx. 580 g/mol *vs.* 3360 g/mol for one end of the PEO segment), resulting thus to a thinner hydrated layer and insufficient steric protection of the newly formed nanocrystal surfaces against aggregation.

In the study, three fundamental requirements for an efficient stabilizer were concluded: firm attachment to the solid surface, high percentage of stabilizer coverage on the nanocrystal surfaces and hydrophilic/lipophilic balance of the stabilizer structure [18]. The hydrophilic/lipophilic balance of the stabilizer structure, *i.e.*, here the balance between PPO and PEO chain lengths, is essential to anchor the block copolymers onto the nanocrystal surfaces. As presented previously, the driving force for adsorption originates from the hydrophobic nature of the PPO segment: the stabilizer molecules are attached via physisorption to drug particle surfaces. Whereas the PEO segments provide the steric hindrance to protect the nanocrystals against aggregation, they simultaneously also prevent, however, the unabsorbed polymer from further adsorbing onto the solid surface, and thus reduce the binding efficacy. In conclusion, with L64 and 17R4, the high affinity and short PEO segments provided poor physical stability due to the thin steric layer. Telechelic structure of 17R4 promoted nanocrystal aggregation due to the molecular bridging between the particles. Linear poloxamer with longer hydrophilic chains did not show as high binding efficiencies, but due to the longer chains for steric stabilization, the overall effect was the best.

Besides affecting the steric layer thickness, molecular weight also affects the viscosity [19]. The approximation for efficient steric stabilization with polymers is that the molecular weight of the polymer should be around 5000–25,000 g/mol for steric hindrance. However, the higher the molecular weight, the higher the viscosity. In the final product, high viscosity can enhance the stability against aggregation, but during the milling or high-pressure homogenization, high viscosity lowers the efficiency of the particle breakage and can also cause blockage of narrow tubes [19,67]. With smaller molecules, like surfactants, the micelle formation may disturb efficient nanocrystal formation by solubilizing the material. With surfactants, it is also important to take into account the CMC value. Below the CMC, monomers tend to attach on the particle surfaces, but above the CMC, micelle formation may be favored. For example, when the amount of pluronic F127 surfactant was increased in order to improve the stability of paclitaxel nanocrystals against aggregation at higher temperatures, the result was the formation of micelles, which lowered the stability of nanocrystals [70]. Desorption experiments showed that, below the CMC, monomers bound strongly to particle surfaces, but above the CMC, the affinity was lower and the surfactant molecules left the particle surfaces. One should also take into account that CMC is temperature related, and at higher temperatures, the CMC value can be much lower. Stabilizers can also function as cryo-/lyoprotectants during freeze-drying [15], and that might be one reason to increase the amount of stabilizer in the composition.

As stated above, poloxamers attach on drug particle surfaces via physisorption. Sometimes, interactions between the functional groups, like phenols, hydroxyls, ethers, amines and carboxylic acids, in drug and stabilizer molecules may cause efficient stabilization [40,44]. Tuomela *et al.* formulated brinzolamide nanocrystals [21]. In this case, physisorption did not take place and poloxamer or polysorbate stabilizers produced only polydisperse microparticles. Instead, HPMC appeared to be a successful stabilizer. Explanation behind the effectiveness of HPMC lies in the molecular structure: HPMC molecules contain a high degree of substitution of the methoxy and hydroxypropoxy groups, which are capable of hydrogen bonding with brinzolamide.

## 4. Effect of Stabilizers on Cell Layers and Drug Transport

**Figure 5:**
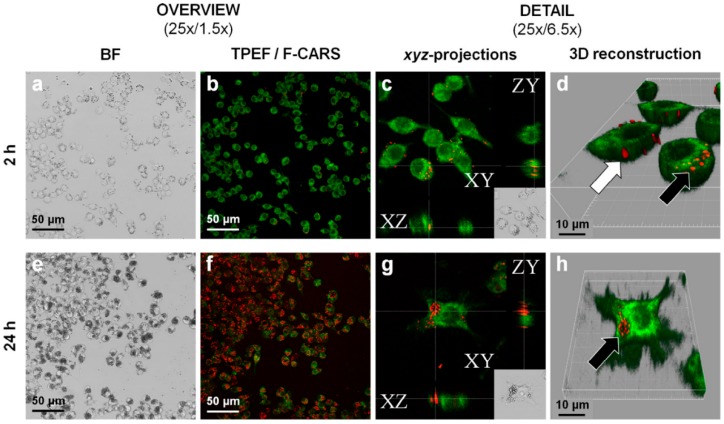
Paliperidonepalmitate nano-/microcrystal–cell interactions with RAW 264.7 macrophages imaged by CARS. Incubation time 2 h (**a**–**d**) and 24 h (**e**–**h**) with 250 µg/mL of paliperidonepalmitate nano-/microcrystals. **a** and **e**: low and high magnification bright field imaging. **b** and **f**: FCARS (**red**)/TPEF (**green**) merged micrographs of stained/fixed RAW 264.7 macrophages. **c** and **g**: orthogonal projections of z-stacked F-CARS/TPEF overlays showing intracellular nano-/microcrystals. **d** and **h**: three-dimensional reconstructions of the z-stacked F-CARS/TPEF overlays. The white arrow shows solid paliperidonepalmitate nanocrystals adsorbed onto the cell surface and black arrows phagocytosed nanocrystals (reprinted from [74] with permission. Copyright Elsevier 2015).

Many stabilizers utilized in drug nanocrystals have some permeation enhancing effect: they can possess transport activities, like, for example, the vitamin E TPGS, poloxamers and polysorbates, which are P-gp inhibitors, or they can open the tight junctions [49]. Gao *et al.* studied TPGS stabilized paclitaxel nanocrystals in the P-gp overexpressing H460 cancer cell line and discovered that TPGS efficiently reduced drug resistance in the cell line [71]. Based on the EPR effect via *iv* administration, drug nanoparticles can be targeted at tumor tissues, although the therapeutic efficacy is lowered by P-gp or other MDR protein activity [72]. Nanoparticles may even be taken up by cells via endocytosis, but when the dissolution takes place in the cytoplasm, P-gp can still pump drug molecules out, if no P-gp inhibitors are present in the cytoplasm simultaneously [73,74] (Figure 5).

If the drug nanocrystals are not immediately dissolved in the GI tract, the adhesion to cell walls can have significant impact on drug bioavailability. Fu *et al.* compared three formulations of lacipidine, namely solid dispersion, micronized drug formulation (particle size 11,200 nm) and nanocrystalline drug formulation (particle size 623 nm) [75]. Both the nanocrystalline and micronized drug were in crystalline form. *In vitro*, the solid dispersion had the highest drug release rate, but *in vivo*, the nanocrystalline product showed a 2.05-fold increase in the AUC-value compared to the solid dispersion. HPMC, which is known to be a mucoadhesive polymer, was used as a stabilizer for the nanocrystals and the micronized drug. The higher AUC value of nanocrystals was due to the adhesion of nanocrystals into the intestinal wall.

After fast dissolution of nanocrystals, the apparent solubility is higher than the thermodynamic solubility, and precipitation may occur. In oral drug delivery, this is extremely important to take into account, especially if the drug material is basic in nature, like itraconazole. In a study by Sarnes *et al.* [5], after fast dissolution, itraconazole was precipitated in the GI tract, when it moved from the stomach to the intestinal area. The reason for this was that the solubility of itraconazole was almost 250 times higher in the stomach as compared to the intestines. In order to reach high bioavailability *in vivo*, the supersaturated state should be able to be maintained and precipitation hindered or delayed. Certain polymers, like PVP [76,77], methacrylate co-polymers [78], HPMC [79], and hydroxypropyl methylcellulose acetate succinate (HPMC-AS) [79] are known to maintain, at least partly, the supersaturation level. However, if the precipitation enhancer is forming micelles, sometimes the affinity of the drug can be higher to the micelles instead of permeation, which decreases the drug adsorption *in vivo* [80]. Ochi *et al.* formulated meloxicam nanocrystals with polymers helping to maintain the supersaturation as stabilizers [77]. The most efficient polymeric stabilizer was PVP; nanocrystalline meloxicam with PVP as a stabilizer reached an *in vitro* supersaturation number of 13.74, as compared to bulk meloxicam. The *in vivo* *C*_max_ value was 6.7-fold higher and the AUC_0–2_ value 5.0 fold higher compared with bulk meloxicam.

Even the shape of the nanocrystals have an impact on the dissolution and bioavailability. Guo *et al.* studied the effect of nanocrystal shape on *in vivo* bioavailability by formulating rod shaped and spherical nanocrystals [81]. They used lovastatin as a model drug. Rod shaped nanocrystals were produced by sonoprecipitation and spherical nanocrystals by milling. Both of the nanocrystals had the same crystal form and a similar hydrodynamic diameter. *In vitro*, the rod shaped nanocrystals had a higher dissolution rate, and also *in vivo* a higher oral bioavailability. The same kind of behavior had been noticed earlier with ketoconazole [82]. The reason for the differences related to particle shape is that lovastatin is a CYP enzyme substrate and has extensive first pass metabolism after the intestinal uptake. After adsorption, part of the lovastatin is hence metabolized. However, because the rod shaped nanocrystals were dissolved faster, the dissolved lovastatin saturated CYP enzymes and extra dissolved lovastatin molecules could reach plasma without being metabolized, and hence increase the oral bioavailability.

Finally, utilization of proteins as stabilizers may have interesting applications because of their ability to bind membrane proteins in tumor cells, which may enable targeting of nanocrystals with the aid of these stabilizers [83]. Lu *et al.* produced paclitaxel nanocrystals stabilized by HPMC, PVP, PEG, Poloxamers, SDS or polysorbates with an antisolvent precipitation technique [84]. Furthermore, human serum albumin, transferrin or immunoglobulin G were mixed with the nanocrystalline suspensions, and these serum proteins were non-covalently bound to the nanocrystalline surfaces. In this study, the transferrin enhanced the *in vivo* antitumor activity of paclitaxel nanocrystals mostly as compared to the nanocrystals without transferrin. The reference product Taxol had still higher efficacy, but paclitaxel nanocrystals with transferrin demonstrated reasonable tumor inhibitory effect with reduced toxicity, which often limits the use of Taxol.

## 5. Discussion

Nanocrystals are one of the most studied novel drug delivery systems due to their considerably easy production and high drug loading. One critical step in the formation of drug nanocrystals is finding suitable stabilizer(s) for the system. Often, stabilizers are thought to be pharmaceutically inactive excipients, and the selection of the stabilizers is performed keeping in mind only the physical stability, e.g., stability against particle aggregation.

However, these stabilizers are not inactive. Many polymers and surfactants utilized as stabilizers for drug nanocrystals are affecting the cells and cell layers. They can, for example, open up the tight junctions or make the cell layers more leaky. Many stabilizers also have impact on active transport systems, like being P-gp inhibitors. They can also have mucoadhesive properties and lengthen the residence time in a certain area of the body. Of course, if the transport activity or mucoadhesion of stabilizers are to be utilized for reaching higher bioavailability, these excipients should be simultaneously present with the drug material in the absorption area. An example of this is paclitaxel formulations with Vitamin E TPGS, a P-gp inhibitor. When paclitaxel in solution was given together with TPGS, the drug was pumped out from the cells by the P-gp because the TPGS was not inside of the cells at the same time [85], while with TPGS stabilized paclitaxel nanocrystals, no P-gp acitivity was seen because of TPGS inhibition [72].

Drug dissolution from nanocrystals produces apparent solubility levels which are above the thermodynamic solubility value of the drug, meaning that the supersaturated state is reached. The supersaturated state is a high energy state, and its equilibrium can be disturbed easily to end up unwanted and fast drug precipitation; for example, pH changes or changes in ionic composition *in vivo* may cause precipitation. In addition, it is worth remembering that after fast dissolution of drug nanocrystals, a drug is in solution, which affects the transit time in the GI tract. Accordingly, fast dissolution *in vitro* is not a guarantee for higher bioavailability *in vivo*. When predicting the *in vivo* behavior of nanocrystals from *in vitro* results, one should be very careful, and deep understanding of the formulation factors are needed.

Stabilizers can also function in some other roles as excipients, for example as a cryoprotectant or disintegrant. This makes it even more important to also think carefully beforehand about the aims and the final formulation. Formation of nanocrystals is only a way to manipulate the raw drug material, and after the nanocrystal production, final formulation is still needed. Molecular engineering of stabilizer molecules may open up new possibilities, when stabilizers can further be functionalized, for example, for targeting purposes or for diagnostics.

A lot of research is heading towards formation of drug nanocrystals, but more emphasis should be put on careful planning of the final drug formulations. The aim should be finding functioning drug delivery platforms, where the drug nanocrystals process step and stabilizer selection are crucial parts of the whole formulation process.

## Figures and Tables

**Table 1:** Examples of drug/stabilizer combinations in nanocrystal formulations.

Drug	Stabilizer	Process	Ref.
Glibizide	Sodium lauryl sulfate, polyvinyl pyrrolidone K30, Pluronics F68 and F127, Tween 80, hydroxypropyl methylcellulose	Milling, antisolvent precipitation	[10]
MTKi-327	Pluronic F108, Lipid S75	Milling	[11]
Beclomethasone dipropionate	Hydrophobin	Antisolvent precipitation	[12]
Naproxen	Vitamin E tocopherol polyethylene glycol succinate, Pluronic F127, sodium lauryl sulfate, di(2-ethylhexyl) sulfosuccinate	Milling	[13]
Paclitaxel	Hydroxypropyl methylcellulose, polyvinyl pyrrolidone, polyethylene glycol 400, Pluronics F127 and F68, sodium lauryl sulfate, Tween 20 and 80, transferrin, Immunoglobulin G, Human serum albumin	Antisolvent precipitation + sonication	[14]
Indomethacin	α-, β- and γ-cyclodextrins	Emulsion solvent diffusion	[15]
Indomethacin	Pluronic F68	Milling	[16]
Budesonide	Lecithin, Pluronic F68	Milling	[17]
Indomethacin	Pluronics F68, 17R4 and L64, Tetronics 908 and 1107	Milling	[18]
Curcumin	Polyvinyl alcohol, polyvinyl pyrrolidone, Vitamin E tocopherol polyethylene glycol succinate, sodium lauryl sulfate, carboxymethylcellulose sodium	High pressure homogenization	[19]
Nitrendipine	Polyvinyl alcohol	Antisolvent precipitation–ultrasonication	[20]
Brinzolamide	Tween 80, Pluronics F68 and F127, hydroxypropyl methylcellulose	Milling	[21]
Fenofibrate	Hydroxypropyl methylcellulose, Soluplus	Milling	[22]
Loviridine, itraconazole, cinnarizine, griseofulvin, indomethacin, mebendazole, naproxen, phenylbutazone, phenytoin	Polyvinyl pyrrolidone, polyvinyl alcohol-polyethylene glycol, Pluronic F68, tocopherol polyethylene glycol succinate, hydroxypropyl methylcellulose, hydroxyethyl cellulose, hydroxypropyl cellulose, methylcellulose, carboxymethylcellulose sodium, polyvinyl alcohol, sodium alginate, Tween 80	Milling	[23]
Nimodipine	Pluronic F127, hydroxypropyl methylcellulose	Microprecipitation, high-pressure homogenization	[24]
